# Medium to long-term outcomes of medial patellofemoral ligament reconstruction using the superficial quadriceps versus a hamstring autograft in patellar instability patients

**DOI:** 10.1038/s41598-023-40610-x

**Published:** 2023-08-16

**Authors:** Prakasit Sanguanjit, Possawat Rujiraphum, Adinun Apivatgaroon, Bancha Chernchujit

**Affiliations:** Department of Orthopaedics, Faculty of Medicine, Thammasat University, Khlong Nueng, Pathum Thani Thailand

**Keywords:** Diseases, Medical research

## Abstract

Medial patellofemoral ligament (MPFL) reconstruction is a standard procedure in patellar dislocation patients. Superficial quadriceps autografts (QA) and hamstring autografts (HA) are popular graft choices in MPFL reconstruction with the lack of directly compared clinical studies between both methods. A total of 43 patellar instability patients, who underwent QA and HA for patellar stabilization at a specified center between 2012 and 2021, were retrospectively reviewed. 21 QA and 22 HA patients were 52.4% of males and 47.6% females with a median age of 25 years (range 12–58) in the QA group, while 63.6% were females with a median age of 21 years (range 14–58) in the HA group. The mean follow-up period was 46.9 months (range 24–77) in QA and 61 months (range 24–100) in the HA group. At the final follow-up, no complications were observed with either technique. No patients presented with recurrent dislocations after surgery. There was no statistically significant difference in postoperative mean Kujala scores (QA = 94.9+/− 4.1 and HA = 94.2+/− 8.0, *p* = 0.73) or the mean Lysholm scores (QA = 94.1+/− 5.0 and HA = 93.2+/− 7.0, *p* = 0.61).

## Introduction

Patellar dislocations occur at a rate of 5.8 per 100,000 persons. Higher incidence in younger patients (10–20 years), females, and athletic sports participation^1,2^. The laxity or injury of the medial patellofemoral ligament (MPFL), bony abnormalities such as trochlear dysplasia; patella alta; laterally positioned tibial tubercle; or femorotibial malrotation are the cause of the patellar instability^3,4^.

MPFL is a primary restraint of lateral patellar displacement from 0 to 30 degrees of knee flexion and is compromised in at least 80% of patellar dislocations^5–8^. Thus, the treatment of MPFL incompetency or laxity is an essential procedure in surgically managing recurrent patellar dislocation.

MPFL reconstruction is the standard surgical method for treating patellar instability patients with or without additional procedures (distal re-alignment, trochleoplasty, tibial or femoral derotational osteotomy, or lateral retinacular release). The indication for isolated MPFL reconstruction is patellar instability in patients without noticeable bony abnormalities such as; a TT-TG distance less than 20 mm, normal trochlear morphology or Dejour type A dysplasia, and absence of patella alta (Caton-Deschamps index < 1.2)^9^. There are many techniques for MPFL reconstruction. However, there is no gold standard according to procedures, graft choices, and fixation methods in MPFL reconstruction^10–12^.

According to the reconstruction of MPFL, graft choice is of fundamental importance. The graft can be harvested from several donors, either autograft, allograft, or synthetic graft. In the recent systematic review of the subjective patient function outcome scores, Kujala scores demonstrated significant improvements across all graft types composed of hamstring and quadriceps tendon with no significant differences among grafts^10^. However, despite the excellent clinical result, several complications have been reported in hamstring tendon graft use, including patellar fractures and implant breakage^13^.

However, there is a lack of clinical studies directly comparing the superficial quadriceps autografts versus hamstring autografts in the same clinical setting, and the proper graft choice for MPFL reconstruction is still unclear. Therefore, this study aims to direct compare clinical outcomes between superficial quadriceps autografts and hamstring autografts. We also evaluated the medium to long-term follow-up complications between both techniques. The hypothesis of the study was no difference in the functional outcomes and complications related to both surgical techniques.

## Materials and methods

A retrospective comparative study, level of evidence 3, investigated the outcomes of patients undergoing MPFL reconstruction with the superficial quadriceps tendon compared with the hamstring tendon for patellar instability and was conducted between 2012 and 2021. The study was approved by Thammasat University Hospital's ethics committee (MTU-EC-OT-6-214/64). All methods were carried out in accordance with relevant guidelines and regulations. Informed consent was obtained from all subjects and/or their legal guardians.

Strict inclusion criteria were applied to all patients. These include recurrent patellar dislocation patients who failed conservative management for more than six months and isolated MPFL reconstruction without additional instability procedures with a minimum follow-up of 24 months. Exclusion criteria were the patients who had previous surgery on the affected knee, concomitant meniscal or other ligamentous injuries of the knee requiring repair/reconstruction, and articular cartilage erosion greater than Outerbridge grade II. Patellar instability is defined as the condition in which patellar dislocation has to occur twice or patellar instability after an initial dislocation. Preoperatively, all patients were evaluated using the standard clinical knee and patella examination. Before surgery, plain anteroposterior and lateral radiographs and magnetic resonance imaging (MRI) were obtained. Medical history, clinical data, and operative reports were collected retrospectively. Functional outcomes and knee range of motion were evaluated at the final follow-up. Functional outcomes were assessed using the Thai version of Kujala scores^14^ and Lysholm scores^15^.

### Surgical technique

Three experienced orthopedic surgeons performed all surgical interventions. (PS, BC, AA) The surgical technique and graft selection depended on the surgeon’s and patient’s preferences.

### Superficial quadriceps tendon autograft

**Figure 1:**
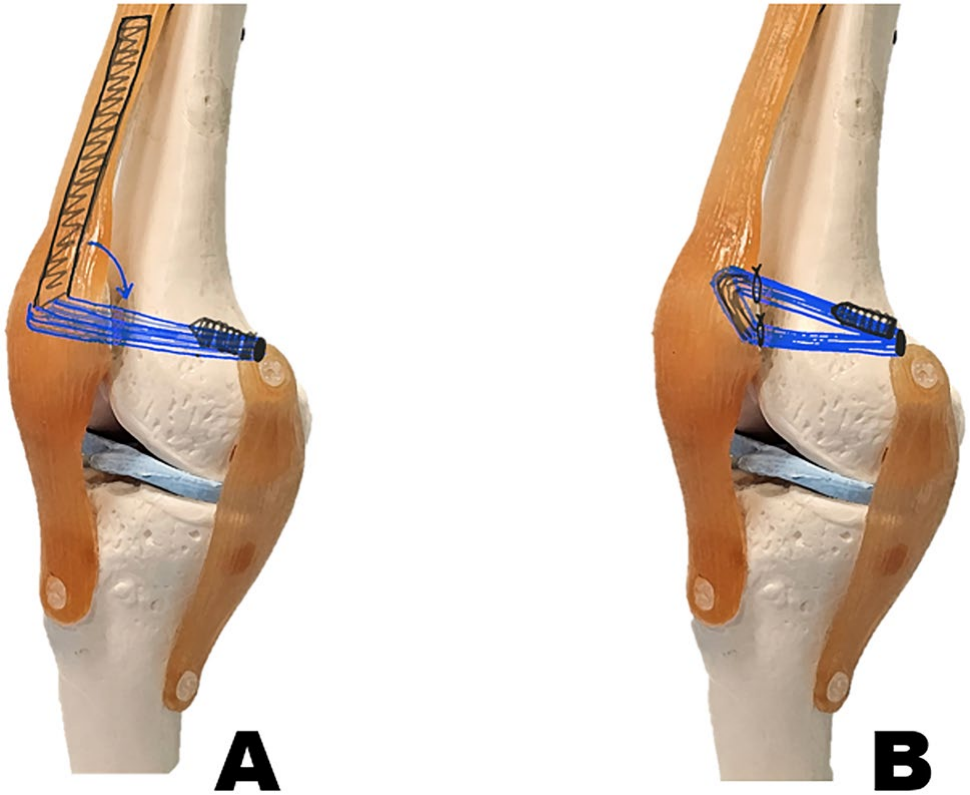
Illustration of the right knee with, (**A**) Superficial quadriceps autograft MPFL reconstruction, (**B**) Hamstring autograft MPFL reconstruction.

The arthroscopic examination was performed as the initial step in conducting a detailed assessment and managing intraarticular injuries. The graft was harvested from the superficial slip of the quadriceps tendon, the rectus femoris tendon^16^. A section approximately 10–12 mm in width was dissected proximally to obtain the required length of 8–10 cm. The proximal edge of the graft was sutured in whip stitch fashion. Subperiosteally dissection of the proximal graft with a sharp knife in an oblique manner, keeping the medial side of the graft intact. The graft was then rotated medially. The femoral fixation point was created using Schottle’s point^13^ with fluoroscopy imaging guidance, and the femoral tunnel was reamed. The graft was passed through the subvastus space to the femoral tunnel from the patellar side. An interference screw 6 × 25 mm (Biosure HA, Smith & Nephew, Andover, MA USA) was inserted with the proper tension at a knee flexion of 45°. Knee arthroscopy confirmed the patella was positioned in the groove and had good tension following reconstruction (Fig. 1A).

### Hamstring tendon autograft

An arthroscopic examination was performed before the MPFL reconstruction to evaluate the intraarticular pathology. The semitendinosus autograft was harvested. The two ends of the hamstring autograft were whip-stitched using nonabsorbable sutures. The graft required a minimum total length of 15–20 cm. A longitudinal incision was made along the medial border of the patella. A 2.0 mm Kirshner wire was inserted at the midpoint of the medial border of the patella through the center. The single patellar tunnel^17^ was created with a 4.5 mm EndoButton reamer (Smith & Nephew, Andover, MA USA). The semitendinosus graft was passed through the patella tunnel with a suture loop. The graft's upper limb was redirected to the superior aspect of the patella and separated from the graft to the upper and lower limbs. The femoral tunnel was created by using Schottle’s point^13^. The two ends of the graft were passed from the anteromedial to the medial side and then through the lateral femoral cortex. An interference screw 6 × 25 mm (Biosure HA, Smith & Nephew, Andover, MA USA) was inserted with the proper tension at knee flexion of 45° (Fig. 1B).

### Postoperative rehabilitation

Following MPFL reconstruction, all patients followed identical rehabilitation protocols^17^. Quadriceps setting and straight leg raising exercises to strengthen starting the day after surgery was encouraged. Full weight-bearing as tolerated with the knee in extension with a knee brace. Range of motion exercises was initiated the day after surgery. Functional activities, including walking, jogging, and running, were introduced at three months postoperatively when patients achieved an acceptable range of motion, muscle strength, and stability. Typically, 4–6 months were needed for patients to return to regular sports activities.

### Statistical analysis

All data were analyzed using SPSS v.28 (IBM Corp.). Categorical data were reported as a percentage. Continuous data were reported as mean, standard deviation, and range (min–max). An a priori power analysis was performed to calculate the sample size required to detect minimal clinically relevant differences in the Kujala scores^18,19^ which we state as the main outcome of our study. The output of the sample size calculation from n4Studies^20^ for testing two independent means formulas. This resulted in a minimum sample size of n = 19 per group (α = 0.05, β = 0.20). The independent T-test was performed in all the comparisons, with values of p < 0.05 considered statistically significant.

## Results

Twenty-one patients in the superficial quadriceps graft group and twenty-two in the hamstring graft group were followed for a minimum of 24 months. 11 males and 10 females with a median age of 25 years (range 12–58) in the superficial quadriceps autograft group. There were 8 males and 14 females with a median age of 21 years (range 14–58) in the hamstring autograft group. The mean follow-up time of the superficial quadriceps group was 46.9 months (range 24–77). The mean follow-up period of the hamstring group was 61 months (range 24–100).

The demographic characteristics of the patients are shown in Table 1.

**Table 1:** The demographic characteristics of the patients. *TT–TG* = Tibial tubercle–trochlear groove. The *p* value was obtained by independent T-test and Chi-square test.

Patient characteristics	Superficial quadriceps (n = 21)	Hamstring (n = 22)	*p* value
Age at surgery (years) Mean (min–max)	26.2 (12–58)	25.9 (14–58)	0.93
Female (%)	47.6	63.6	0.29
Left side (%)	76.2	68.2	
Body mass index (kg/m^2^)	22.8+/− 4.9	24.4+/− 5.8	0.32
Caton-Deschamps ratio	1.09 ± 0.16	1.08 ± 0.17	0.85
TT-TG distance (mm)	16.7+/− 3.9	14.6+/− 3.3	0.06
Follow-up time (months) Mean (min–max)	46.9 (24–77)	61 (24–100)	0.04

There were no differences in pre-operative demographic data regarding age, gender, side of injury, the Caton–Deschamps ratio, and the mean TT-TG distance. In addition, the mean follow-up time in the superficial quadriceps group was less than in the hamstring group (p = 0.04) as the superficial quadriceps MPFL reconstruction technique was developed at a later time than the hamstring graft technique.

The comparable clinical scores (Kujala and Lysholm scores) and knee range of motion for superficial quadriceps autograft and hamstring autograft groups at the final follow-up are presented in Table 2. The mean postoperative Kujala scores were 94.9+/− 4.1 and 94.2+/− 8.0 for the superficial quadriceps and hamstring groups, respectively. There were no statistically significant differences between the two groups.

**Table 2:** Comparison of the knee's clinical scores and range of motion for superficial quadriceps autograft and hamstring autograft groups. The *p*-value was obtained by independent T-test.

Clinical scores	Superficial quadriceps	Hamstring	*p*-value
Kujala score	94.9+/− 4.1	94.2+/− 8.0	0.73
Lysholm score	94.1+/− 5.0	93.2+/− 7.0	0.61
Range of motion (degree)	140+/− 6.7	138+/− 8.5	0.33

No patients suffered from a patellar re-dislocation or subluxation at the final follow-up. No infection or patellar fracture was observed during the follow-up period in any patient. No patients had skin sensation loss around the affected knee after the final follow-up. No statistically significant differences in clinical scores were found between the groups.

## Discussion

The most important finding of this study was that both procedures, superficial quadriceps and hamstring autograft for MPFL reconstruction, are safe and reproducible procedures with good clinical and functional outcomes at minimum, the two years of follow-up. There were no differences in pre-operative demographic data regarding age, gender, side of injury, the Caton-Deschamps ratio, and the mean TT-TG distance. In addition, the mean follow-up time in the superficial quadriceps group was less than in the hamstring group (*p* = 0.04) as the superficial quadriceps MPFL reconstruction technique was developed at a later time than the hamstring graft technique.

The mean postoperative Kujala scores were 94.9+ /− 4.1 and 94.2+/− 8.0 for the superficial quadriceps and hamstring groups, respectively. There were no statistically significant differences between the two groups. The results were compared with previous studies that used superficial quadriceps or hamstring tendons as the graft source^10^. A recent systematic review published by McNeilan et al.^10^ showed that the mean postoperative Kujala score ranged from 84.4 to 94 in quadriceps autograft and 81.7 to 94.6 in hamstring autograft.

A biomechanics study^21^ to evaluate the ultimate failure load and stiffness of quadriceps tendon fixation and single tunnel patella fixation with gracilis autograft in MPFL reconstruction revealed no statistical difference in maximum load to failure and stiffness between both techniques. However, from the biomechanical study with the hamstring group, the patellar fixation method, graft type, and graft orientation technique differed when compared with our reconstruction technique.

The MPFL is the important medial restraint structure during the first 30° of knee flexion^5^. Mountney et al.^22^ stated that the MPFL ruptured at a mean of 26+/− 7 mm, and the dislocated patella 50 mm, ensuring the rupture of MPFL. Graft selection for the MPFL reconstruction is important. The biomechanical properties of the grafts have been investigated. Tensile strength and viscoelastic properties of the reconstructed tendon graft are the critical mechanical parameters for successful reconstruction. The MPFL is a ligament of tissue connecting the tubercle of an adductor tubercle of the distal femur to the proximal aspect of the medial edge of the patella^23^. The native tensile strength of the MPFL is approximately 208 N^19^, both quadriceps and hamstring tendon grafts are more restrained^24,25^. Herbort et al.^24^ found that the tensile strength of the superficial quadriceps tendon is 205 N. The tensile strength of the semitendinosus and the gracilis tendon is 1060 N and 837 N, respectively^26^.

All of the cases in our study's superficial quadriceps and hamstring graft groups use the same technique for femoral fixation. An interference screw fixation at the femoral tunnel using fluoroscopy to locate Schottle’s point^13^. We used fluoroscopic landmarks to confirm the ideal location of the femoral footprint MPFL to avoid failure due to femoral tunnel malposition, which is the common cause of MPFL failure^27^. The appropriate knee flexion angle is still controversial, ranging from 30°, 45°, 60° to 90° of knee flexion from literature. Regardless of the degree of knee flexion, the range of motion following graft fixation should be fully obtained. There should be a suitable endpoint to lateral patellar translation from 0° to 30° of knee flexion, and proper tension confirmed in both techniques.

Despite a high success rate, the combined complication and failure rate of MPFL reconstruction remain substantial, reported at 26% in one systematic review^28^. Complications included graft failure, graft overtightening, and patellar fracture. The patellar fracture was one of the most serious complications reported after MPFL reconstruction with a hamstring graft. The superficial quadricep graft can eliminate complications associated with patellar fracture. The drawbacks of superficial quadriceps graft are increased scar formation at the harvested site and graft amputation during harvesting. However, our series had no reported patellar fractures or superficial quadriceps graft amputations.

This study is the first study to directly compare the clinical outcomes between superficial quadriceps autograft and hamstring autograft in MPFL reconstruction. Many published studies were reported in the case series without comparing groups. This study has some limitations; the first was the wide range of follow-up time points to evaluate clinical outcomes and scores. The second was that both groups were not operated on by a single surgeon with different surgical techniques, which may result in a bias in clinical examination and clinical outcomes. The graft selection depended on the surgeon’s and patient’s preferences, leading to selection bias. The third was that the small sample size may have affected our finding that there was no difference in outcomes between the two groups. Fourth, no data on the preoperative Kujala and Lysholm scores due to a retrospective basis. Lastly, the donor site morbidity of the quadriceps and hamstring strength or functions was not evaluated post-operatively.

## Conclusion

MPFL reconstruction for patellar instability surgery using either superficial quadriceps or hamstring autograft achieved good clinical outcomes during a minimum follow-up of 2 years. No significant differences in clinical outcomes and complications were observed in both groups.

## Data Availability

The datasets used and/or analyzed during the current study are available from the corresponding author upon reasonable request.
